# Iodine intake and status of school-age girls in Ireland

**DOI:** 10.1007/s00394-025-03731-9

**Published:** 2025-06-09

**Authors:** Emma Kane, Maria Buffini, Aoibhín Moore Heslin, Anne P. Nugent, Laura Kehoe, Janette Walton, John Kearney, Albert Flynn, Breige McNulty

**Affiliations:** 1 UCD Institute of Food and Health, University College Dublin, Dublin, Ireland; 2 Institute for Global Food Security, Queen’s University Belfast, Belfast, UK; 3 School of Food and Nutritional Sciences, University College Cork, Cork, Ireland; 4 Department of Biological Sciences, Munster Technological University, Cork, Ireland; 5 School of Biological, Health and Sports Sciences, Technological University Dublin, Dublin, Ireland

**Keywords:** Iodine, Children, Teenagers, Females, Ireland

## Abstract

**Purpose:**

Insufficient iodine intake can lead to a variety of preventable health and developmental outcomes. Consequently, continuous monitoring of a population’s iodine adequacy is important. The aim of this study was to examine iodine intake and status in a nationally representative sample of school-age girls living in Ireland.

**Methods:**

Analyses were based on 516 schoolgirls aged 5–18 years from the cross-sectional National Children’s Food Survey II (2017–2018) and the National Teens’ Food Survey II (2019–2020). Dietary data were collected using a four-day weighed food diary. Adequacy of iodine intake and the contribution of food categories to overall dietary iodine intake was assessed. Urinary iodine concentration (UIC) was measured using a spot urine sample using the Sandell-Kolthoff reaction by a modified microplate method.

**Results:**

Median iodine intake was 94.8 µg/d (IQR 54.9–155.5 µg/d), with 40% of girls having intakes below the estimated average requirement (EAR). The median UIC was 87.8 µg/L, with younger girls having a significantly higher UIC compared to older girls (104.4 µg/L − 77.0 µg/L respectively; *p* < 0.001). The main dietary source was milk, contributing to over 50% of iodine intake, with non-consumers of milk having significantly lower dietary and urinary iodine levels compared to high consumers (*p* < 0.001).

**Conclusion:**

This study suggests that school aged girls living in Ireland have mild iodine deficiency and given the concerns associated with such deficiencies and their potential public health implications, it is essential that iodine status in other population groups in Ireland is ascertained. The need for targeted public health strategies to eradicate iodine deficiency may be warranted.

**Supplementary Information:**

The online version contains supplementary material available at 10.1007/s00394-025-03731-9.

## Background

Iodine is an essential trace mineral that plays a significant role in metabolism throughout our lifespan, and in growth and neurodevelopment in utero and during childhood [1, 2]. This essential mineral is a main component of the thyroid hormones, thyroxine (T4) and triiodothyronine (T3), which are produced and regulated from the thyroid gland and are involved in various metabolic processes such as protein, fat, and carbohydrate metabolism [3]. Inadequate iodine can lead to adverse health effects, the collective term for which are known as Iodine Deficiency Disorders (IDD) [4]. Conditions such as hypothyroidism, cretinism, endemic goitre, and endemic mental retardation can occur due to a severe deficiency of iodine [5]. However, these conditions rarely now occur in Western populations, mainly due to the influence of iodine-containing compounds used in irrigation, fertilizers, and livestock feed, good availability of a diverse range of foods in Western populations and the introduction of mandatory iodised salt programmes, where salt used in households and in food processing is routinely fortified with iodine to prevent iodine deficiencies.

Severe deficiencies of iodine are uncommon in high-income countries (HIC) countries, however, mild-to-moderate iodine deficiencies are of more concern on account of the growing prevalence in many HIC [6, 17]. Research suggests that mild-to-moderate iodine deficiency during pregnancy may negatively impact cognitive function of the offspring [7–11]. Additionally, some longitudinal studies suggest that a low habitual intake of iodine during the gestation period is associated with increased child ADHD symptoms at 8 years of age [10] and poor language skills, school performance and greater likelihood of requiring special educational support at 8 years of age [12]. Furthermore, low maternal urinary iodine during pregnancy has been associated with differences in memory function and auditory processing speed [9, 13], substandard spelling [8] and suboptimal cognitive function in the reading abilities of 8-year-old children after adjusting for possible confounders [7].

Due to the consequences that iodine deficiency can cause in a population, the World Health Organization (WHO) recommends that iodine status is measured every five years at a population level [2]. Continuous monitoring of a population’s iodine adequacy is important, especially in populations that are vulnerable and borderline sufficient. It is known that iodine intake can change over time due to changes in the food supply, nutrition policies and regulations, and changing dietary habits. Thus, it is important that iodine intake and status are assessed regularly. In order to evaluate iodine status in a population, it is advised that urinary iodine concentration (UIC) from spot urine samples in children aged 6–12 years are used, the reasoning being that the school setting offers easy accessibility in terms of sample collection. In addition, in terms of adequacy, the WHO have suggested that a median UIC of 100–199 µg/L indicates that the population has adequate iodine nutrition [2, 14]. This range applies to children and adults and excludes pregnant and lactating women.

For many years, the UK and Ireland were thought to have sufficient iodine nutrition due to changes in dairy farming practices which led to the contamination of milk with iodine and encouragement of milk consumption post war which contributed to the eradication of iodine deficiency in the 1930s [15]. More recently however, there has been concern as although evidence indicates levels are adequate based on previous studies in Irish adults conducted in 2008-10 [16] and in adolescent females conducted in 2014-15 [17, 18], the UIC reported were just slightly greater than the 100 µg/L cut-off (107 µg/L and 111 µg/l respectively). Furthermore, changes in dairy consumption patterns in school-age children (2017–2018) and adolescents (2019–2020) living in Ireland have been noted, with milk consumption declining in recent years [19, 20]. As there is no iodised salt programme or iodine fortification programme in Ireland, and with dairy known to be the major source of iodine in Ireland, this decrease in milk consumption and possible impact on iodine status is cause for concern. This is particular pertinent to young females as available data suggest they are vulnerable to iodine deficiency [18]. Therefore, this analysis aims to examine the iodine intake and status in a nationally representative sample of school-age girls living in Ireland and to investigate the dietary and sociodemographic factors that may be associated with iodine adequacy.

## Methodology

### Participants and data

Data used for analyses were obtained from two cross-sectional food consumption surveys: the National Children’s Food Survey II (NCFS II; 2017–2018; 5–12 years of age) and the National Teens’ Food Survey II (NTFS II; 2019–2020 13–18 years of age). Only female participants from both surveys were included in this analysis (*n* 516). Ethical approval was granted from the University College Cork (UCC) Clinical Research Ethics Committee of the Cork Teaching Hospitals and the Human Ethics Research Committee of University College Dublin (UCD) for both the NCFS II (ECM 4 aa 07/02/2017) and the NTFS II (ECM 4 (II) 04/12/2018). In line with the Declaration of Helsinki guidelines, parents/guardians of the children provided informed written consent for the NCFS II, and both the adolescent and their parent/guardian gave written assent and consent, respectively, for participation in the NTFS II. The overall response rate was 65% for the NCFS II and 57% for the NTFS II. Further information on the sampling procedures and the survey methodologies has been published elsewhere [19, 20]. In brief, both surveys were nationally representative in terms of age-group, sex, and geographical location in relation to the Irish census data [21]. However, as there were a higher number of participants with a higher social class compared to the census data a statistical weighting factor was applied.

### Assessment of iodine intake

A consecutive four-day weighed food diary, inclusive of at least one weekend day, was used to collect food and beverage consumption and supplement use for female participants in both the NCFS II and NTFS II. The combined database on school-age girls produced 32,213 rows of data, which included 2190 food and beverage codes. Dietary iodine intake (µg/day) was estimated using food iodine composition data obtained from the 2012–2014 Irish Total Diet Study (TDS) [22]. Each food, beverage, and recipe were allocated a mean iodine concentration value (µg/100 g) based on the Irish TDS data (95.6%). When data were not available or suitable from the Irish TDS database, manufacturer’s information was used for brand-specific products (1.3%) and online national food composition databanks were used which included the Finnish Institute of Health and Welfare national food composition database [23] (1.8%), the UK McCance and Widdowson’s Composition of Foods Integrated Dataset [24] (< 1%), the United States Department of Agriculture (USDA) Food Data Central database [25] (< 1%) and the Food Standards Australia and New Zealand food composition database [26] (< 1%). The iodine contents of recipes were calculated by disaggregating the recipe and determining the iodine content of each individual ingredient, which were then summed to give the total iodine content of each recipe. The seasonal variance of milk (including whole milk, semi-skimmed milk, skimmed milk, and fortified milks) on iodine intake was estimated, by applying an iodine concentration based on the season in which the participant took part in the survey, which was assigned using data from the TDS [22]. Food and beverage codes were categorised into one of twenty-four iodine-specific food categories [16]. Iodised salt was not considered in the dietary assessment as Ireland does not have a salt iodisation programme, and it is uncommon to find iodised salt in Irish supermarkets [73].

### Recommended iodine intake

Adequacy of dietary iodine intake were compared to national and international recommendations. The reference values used for this analysis included the Lower Reference Nutrient Intake (LRNI) (50–70 µg/d) from the UK Department of Health (DOH) [27], the Estimated Average Requirement (EAR) (65–95 µg/d) from the US Institute of Medicine (IOM) [28], and the Tolerable Upper Level (TUL) (250–600 µg/d) of iodine derived from the European Food Safety Authority (EFSA) [29]. All reference values were age-specific. Under-reporters were included in this analysis as exclusion of under-reporters did not indicate differences in iodine intake. To identify energy under-reporters, Black’s updated version of Goldberg’s cut-off was used [30–32]. Overall, 33% of school-age girls were classed as under-reporters using these criteria.

### Urinary iodine concentration sampling and analysis

Children and adolescents who took part in the NCFS II and NTFS II were asked to provide a fasting first void spot urine sample, this was provided during the four day dietary collection period. Urine samples were collected during all months and seasons of the survey periods. Samples were stored on dry ice during transportation to laboratories in Dublin or Cork where they were then stored at − 20 °C until batch processing. UIC was measured colourimetrically using the Sandell-Kolthoff reaction by a modified microplate method and standard operating procedures in accordance with the Centers for Disease Control and Prevention (CDC) Ensuring the Quality of Iodine Procedures (EQUIP) program protocol [33, 34]. The nutrition lab in University College Dublin was used to complete all UIC measurements and was registered with the EQUIP quality assurance programme (CDC Atlanta, GA, USA). Blind analysis and quality control measures in the form of repeat analysis of pooled samples were carried out. Inter- and intra-assay CV were 14.1% and 4.0% respectively. The limit of detection was ≤ 10 µg/L.

### Sociodemographic and lifestyle factors

For these analyses, participants were divided into two age-groups 5–10-year-olds and 11–18-year-olds to obtain an even distribution while also taking into consideration that the WHO states that the adolescence period begins before the age of 13 [35]. Anthropometric measures were taken by the researchers. Height was measured to the nearest 0.1 cm using the Leicester portable height measure (Seca, Birmingham, UK) with the participant’s head positioned in the Frankfurt Plane. Weight weas measured (in duplicate) to the nearest 0.1 kg using a Tanita body composition analyser BC-420MA (Tanita Ltd, GB). Participants were weighed after having voided, wearing light clothing and without shoes. Body Mass Index (BMI) was calculated by dividing weight (kg) by height squared (m^2^). As age and sex-specific BMI charts are lacking in Ireland for the Irish population, the International Obesity Task Force (IOTF) age and sex-specific cut off values were used to classify participants into BMI groups [36]. Household location was classed as rural (≤ 1500 habitants) or urban (> 1500 habitants) in line with the 2016 Census [21]. Highest reported parental occupation was used to determine the participant’s social class group. The four social class groups are listed in descending order and include (1) Professional, managerial, and technical workers, (2) Non-manual workers, (3) Skilled manual workers, and (4) Semi-skilled and unskilled workers [21]. Parent’s/guardian’s highest level of education attained to date was classed into one of two categories: secondary education and lower, or tertiary education [21]. Similar to the method used for the classification of social class, the parent with the highest education level data were used if parents/guardians achieved two different levels of education.

### Dietary factors

To assess whether supplement use had an associated with iodine intakes, information on general supplement use was split into two categories ‘yes’ or ‘no’ based on whether the participant reported consuming any nutritional supplement within the past 12 months. This classification was not specific to iodine containing supplements, it encompassed the use of any type of nutritional supplement. Overall, 28% of girls reported consuming at least once nutritional supplement within the previous 12 months. Mean daily energy intake was estimated for each participant along with the percentage of under-reporters based on their energy intake, as discussed above. Total milk consumption which includes whole, low fat, skimmed and fortified milk was calculated for each participant to assess the relationship between and milk and iodine intake and status. Plant-based milk alternatives were not included in the analysis as consumption was reported by less than 5% of participants and typically contained minimal or no iodine.

### Statistical analyses

Statistical analysis was carried out using the IBM SPSS^©^ software (version 27 SPSS, Inc., Chicago, IL, USA). The data used in this analysis was positively skewed, thus, the results were presented as median and interquartile ranges (IQR). Differences between age groups and iodine intake were assessed by a Mann-Whitney U test or by a Kruskal-Wallis test. Median and IQR were also used to present UIC data and differences between age groups and season were assessed by a Mann-Whitney U test. Season was also divided into two groups: October-March (winter) and April-September (summer). The percentage contribution of 24 specified food categories to iodine intake were estimated and subsequently split by age group (5–10 years vs. 11–18 years). Statistical significance between the two age groups across food categories was determined using an independent *t* test.

To assess the association between milk consumption and iodine intake and UIC, participants were grouped into four categories based on their milk consumption patterns. The four groups comprised of non-consumers which included participants who reported consuming no milk during the survey (0 g/day), low consumers (2–87 g/day), medium consumers (88–192 g/day), and high consumer of milk (193–845 g/day). Consumers of milk were grouped into low, medium and high based on their mean daily consumption of milk. Covariate-adjusted univariate general linear models, including age, household location, education level of parent/guardian, social class, and underreporting as covariates, were used to assess differences between iodine intake and status with milk consumption habits.

Univariate and backward multivariable linear regression analysis was performed to determine the sociodemographic and dietary factors that may be associated with iodine intake. Standardised beta coefficients, unstandardised coefficients and 95% confidence intervals are presented for all linear regression analyses. A *p* value of < 0.05 denoted statistical significance for all statistical analyses. Effect sizes are indicated for significant results as partial eta squared (n^2^p), with n^2^*p* < 0.06 deemed as a small effect, n^2^*p* = 0.06 < 0.14 deemed as a medium effect and n^2^*p* ≥ 0.14 classed as a large effect [37].

## Results

### Study population characteristics

In total, 516 school-age girls (aged 5–18 years) were included in these analyses, of these 96% provided a urine sample. The average age of the population was 11.3 years, and the mean BMI was 19.5 ± 4.3 kg/m². Over 90% of the population were of white ethnicity and 60% of the population lived in an urban area. The majority of parents/guardians attained a tertiary education (84%) and were classified into the highest social class group; professional/managerial/technical (63%).

### Iodine intake in school-age girls

Median iodine intake was 94.8 µg/d (IQR 54.9–155.5 µg/d) (Table 1). Younger girls aged 5–10 years had significantly higher iodine intake in comparison to older girls aged 11–18 years (108.4 v 86.0 µg/d; *p* < 0.001). In the total population, those living in a rural location had higher iodine intakes compared to those living in an urban location (101.9 v 89.3 µg/d), this was also observed in the older group (11–18 years) but not for younger girls (5–10 years). Girls whose parents/guardians had a lower level of education and social class had significantly greater iodine intake compared to girls whose parents attained a tertiary level of education (96.9 v 92.5 µg/d) and had a higher social class (136.7 v 77.5 µg/d). The differences between social class groups persisted after splitting by age group with those in the highest social class group having the lowest iodine intake across both age groups. There were no significant differences noted between BMI groups or between supplement users.

**Table 1:** Median iodine intakes (µg/d) of school-age girls living in Ireland split by age group across sociodemographic and lifestyle characteristics

			Total Population (5–18 years) (*n* 516)			Younger Girls (5–10 years) (*n* 226)			Older Girls (11–18 years) (*n* 290)		
			Median	IQR		Median	IQR		Median	IQR	p value*
All Girls			94.8	54.9, 155.5		108.4	62.6, 181.4		86.0	51.0, 135.0	< 0.001
BMI											
	Normal weight		95.1	57.7, 153.5		110.5	63.6, 173.1		84.6	50.4, 133.7	< 0.001
	Overweight/obesity		93.2	54.0, 156.9		98.8	58.1, 193.1		91.3	53.8, 152.2	0.274
Location											
	Rural		101.9^a^	64.1, 168.1		109.9	67.0, 191.7		97.0^a^	62.3, 159.5	0.344
	Urban		89.3^b^	49.9, 143.7		104.9	59.8, 163.9		82.9^b^;	47.9, 125.3	< 0.001
Education level of the parent/guardian											
	Secondary & lower		96.9^a^	62.8, 175.1		127.8^a^	70.5, 255.2		90.1	52.2, 158.4	0.042
	Tertiary		92.5^b^	53.9, 149.3		104.9^b^	58.7, 169.9		84.9	49.6, 133.3	< 0.001
Social class†											
	Professional/managerial/technical		77.5^a^	45.7, 125.9		89.4^a^	49.4, 137.5		70.2^a^	39.9, 111.1	0.005
	Non-manual skilled		110.6^b^	69.3, 169.9		123.0^b^	82.9, 211.5		99.5^b^	62.6, 157.2	0.077
	Manual skilled		103.8^b^	66.7, 172.9		157.6^b^	71.7, 265.2		86.6^b^	54.1, 155.5	0.020
	Semi-skilled/unskilled/students		136.7^c^	91.3, 257.2		225.1^b^	113.9, 316.3		121.8^b^	87.3, 168.8	0.037
Supplement user											
	Yes		98.0	51.8, 157.6		100.7	59.0, 158.7		95.7	49.3, 156.1	0.260
	No		91.7	55.7, 151.5		112.2	62.8, 193.1		84.7	51.4, 130.2	< 0.001

^**a, b, c**^ Median values with unlike superscript letters are significantly different between groups (*p* < 0.05). Differences between age groups, BMI, location, education level of the parent/guardian and supplement users were assessed by a Mann-Whitney U test. † Differences across social class groups were assessed by a Kruskal-Wallis test.* Differences across age groups within the same socio-demographic grouping were assessed by a Mann-Whitney U test. Social class weighting factor applied to data. BMI, body mass index; IQR, interquartile range

In this cohort, 28% of girls had intakes less than the LRNI (Table 2), with slightly more 11–18-year-olds (32%) having intakes less than the LRNI compared to the 5–10-year-old females (25%). In terms of the EAR, 40% of the total population did not meet this recommendation respectively. The same trend was observed in relation to age group with 48% of 11-18-year-olds versus 34% of 5–10-year-olds failing to meet the EAR for their age group. Regarding upper levels of intake, 5% of the total population exceeded the TUL, with this being more notable in the younger cohort (8% of 5–10-year-olds compared to < 1% of 11–18-year-olds), however it should be noted that the TUL is at a lower cut-off in the younger cohort. Further analysis also indicated that milk was a major contributor to those who exceeding the TUL.

**Table 2:** Percentage of school-age girls living in Ireland not meeting recommended iodine intakes

	*n*	% below LRNI µg/d*	% below EAR µg/d†	% above UL µg/d§
Total Population (5–18 years)	348	27.9	40.2	4.9
Younger Girls (5–10 years)	196	24.5	34.2	8.2
Older Girls (11–18 years)	152	32.2	48.0	0.7

LRNI, lower reference nutrient intake; EAR, estimated average requirement; UL, tolerable upper level. * Department of Health [26] (50–70 µg/d) † Institute of Medicine [27] (65–95 µg/d) § European Food Safety Authority [28] (250–600 µg/d)

### Urinary iodine concentrations

The median UIC for the total population was 87.8 µg/L (IQR 52.3-136.5), with 5–10-year-old girls having a significantly higher UIC of 104.4 µg/L compared to 77.0 µg/L for 11–18-year-old girls (*p* < 0.001). A significant positive correlation was noted between UIC and dietary iodine intake (R_s_ 0.361; *p* < 0.001). The UIC was higher in those surveyed during the autumn/winter months compared to those surveyed in the spring/summer months (93.2 vs. 83.5 µg/L; *p* = 0.041) and a similar trend was noted across both age groups with higher UIC for the autumn/winter months (Table 3).

**Table 3:** Urinary iodine concentrations in school-age girls living in Ireland spilt by age group and season

	Total population			Age groups						
	All (5–18 years)			Younger Girls (5–10 years)			Older Girls (11–18 years)			p value*
	*n*	Median	IQR	*n*	Median	IQR	*n*	Median	IQR	
UIC (µg/L)										
All	496	87.8	52.3, 136.5	216	104.4	65.5, 169.0	280	77.0	53.1, 188.6	< 0.001
Season										
Oct-Mar	283	93.2^a^	58.6, 146.9	141	106.0^a^	65.0, 187.6	142	79.3^a^	56.6, 120.5	< 0.001
Apr-Sept	213	83.5^b^	53.3, 122.4	75	99.2^b^	66.2, 149.2	138	73.7^b^	48.1, 117.3	0.005

* Differences between age groups and seasons were assessed by Mann-Whitney U tests. ^a^,^b^ Different lower case superscript letters indicate statistical significance between seasons (*p* < 0.05). IQR, interquartile range

### Percentage contribution of food categories to iodine intake

The main dietary contributor of iodine for this cohort of school-age girls was milk (whole, low fat, skimmed and fortified milk), which contributed to over 50% of dietary iodine intake (Table 4). The next biggest contributors were breakfast cereals, creams, ice creams and desserts, and grains, rice, pasta, and savoury dishes, each contributing 4–5% to dietary intake. Younger girls obtained significantly more iodine from whole milk, breakfast cereals, and yogurts than their older counterpart whereas older girls had a significantly higher contribution of iodine from savoury dishes, beverages, and chocolate confectionery than younger girls. It was also noted that older girls (11–18 years) consumed significantly less milk (including whole, semi-skimmed, skimmed, and fortified milks) in comparison to younger girls aged 5–10 years (112.6 g/d vs. 165.3 g/d, respectively) (*p* < 0.001) (Online Supplementary Fig. S1).

**Table 4:** Percentage contribution of food and beverage categories to intakes of iodine split by age group (mean values and standard deviations)

Food Categories	% consumers of food category	Total Population (5-18yrs)	Younger Girls (5-10yrs)	Older Girls (11-18yrs)	*p* value*
Beverages	99	2.8	1.3	4.1	< 0.001
Bread & rolls	97	1.1	0.9	1.3	0.211
Meat & meat products	97	2.2	1.8	2.5	0.110
Grains, rice, pasta & savouries	92	4.1	2.6	5.4	< 0.001
Fruit & fruit dishes	89	1.4	1.3	1.4	0.650
Vegetable & veg dishes	89	0.6	0.4	0.7	0.003
Potatoes & potato dishes	89	0.6	0.5	0.7	0.014
Biscuits, cakes, & pastries	85	2.0	1.9	2.2	0.858
Breakfast cereals	82	5.0	6.7	3.5	< 0.001
Soups, sauces & miscellaneous foods	82	1.0	0.6	1.4	0.030
Sugars, preserves & savoury snacks	82	0.6	0.5	0.7	0.348
Butter, spreading fats & oils	80	0.4	0.5	0.4	0.062
Cheeses	62	2.4	2.3	2.4	0.511
Creams, ice-creams and desserts	60	4.3	4.2	4.3	0.465
Whole milk	58	36.4	43.0	30.5	0.004
Chocolate confectionery	58	3.6	2.7	4.3	0.015
Yogurts	48	3.6	4.7	2.6	< 0.001
Non-chocolate confectionery	48	0.2	0.1	0.3	0.026
Fish & fish dishes	38	2.8	2.6	2.9	0.866
Eggs & egg dishes	36	4.0	3.4	4.5	0.446
Low fat, skimmed & fortified milks	30	15.1	13.4	16.6	0.819
Other milk & milk-based beverages	21	3.7	3.2	4.2	0.645
Nutritional Supplements	19	2.2	1.2	3.1	0.159
Nuts, seeds, herbs & spices	16	0.0	0.0	0.0	0.003
Total (%)		100	100	100	

* Differences between age groups were assessed by an independent *t* test. Yrs, years

### Relationship of milk with iodine intake and status

**Fig. 1:**
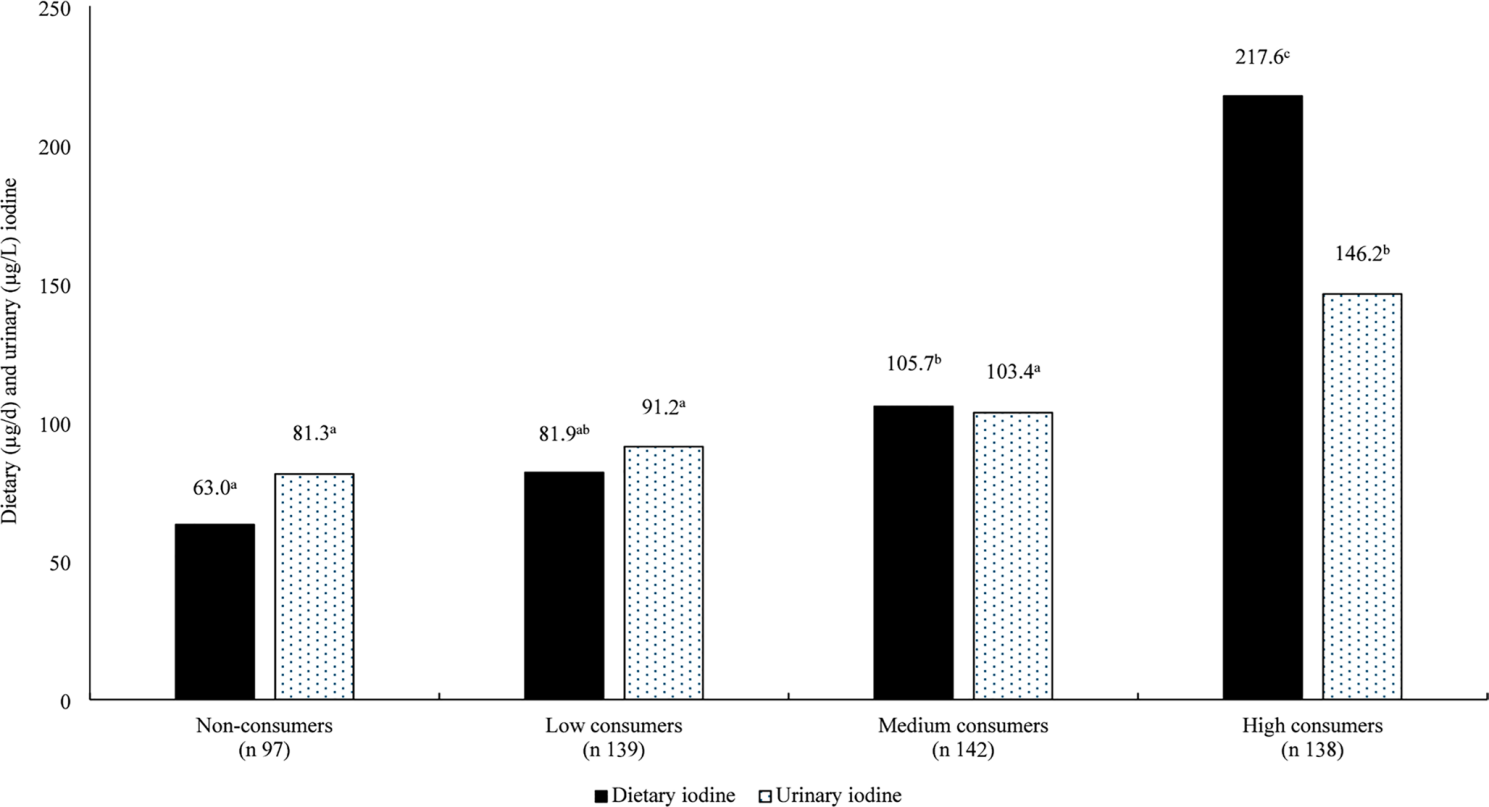
The relationship between quartiles of milk consumption with dietary iodine intake (µg/d) and urinary iodine status (µg/L) Milk intake used to calculate consumption groups refers to consumption of whole, semi-skimmed and skimmed milks; non-consumers recorded no consumption of milk (0 g/d), low consumers (2–87 g/d), medium consumers (88–192 g/d) and high consumers (193–845 g/d). Data presented as adjusted means derived from a covariate-adjusted general linear model. Covariates included: age, household location, education level of parent/guardian, social class, and underreporting. ^a^,^b^,^c^ Mean values with different lower case superscript letters were significantly different. Dietary iodine *p* < 0.001, n^2^*p* = 0.238; Urinary iodine *p* < 0.001, n^2^*p* = 0.094

As milk was a major contributor to iodine intake, milk consumers were split into non-consumers and tertiles of consumers, with iodine intake and status assessed across these groups (Fig. 1). Non-consumers of milk had significantly lower iodine intake and status compared to high consumers of milk after adjusting for the covariates age, location, education level of parent/guardian, social class, and underreporting (*p* < 0.001).

### Sociodemographic and dietary factors associated with iodine intake

Univariate and backward multivariable linear regression analysis indicated that the strongest factors that positively associated with iodine intake were milk consumption (β = 0.617; *p* < 0.001) and a lower social class (β = 0.253; *p* < 0.001), followed by a higher energy intake (β = 0.101; *p* = 0.002) and a lower level of paternal education (β = 0.083; *p* = 0.011). Living in an urban location had a negative relationship with iodine intake however the association was weak (β = -0.065; *p* = 0.041) (Table 5).

**Table 5:** Univariate and backward multivariable linear regression assessing sociodemographic and dietary factors with iodine intakes for school-age girls

Sociodemographic & dietary factors	Univariate model			Multivariable model		
	Dietary iodine (µg/d) (*n* 516)			Dietary iodine (µg/d) (*n* 516)		
	β	B (95% CI)	*p value*	β	B (95% CI)	*p value*
11–18-year-old age group	-0.092	-19.751 (-38.7, -0.8)	0.041	-0.001	-0.108 (-18.3, 18.1)	0.991
BMI (kg/m²)	-0.008	-0.252 (-3.0, 2.5)	0.859	-	-	-
Lower parental education level	0.151	54.971 (23.6, 86.3)	< 0.001	0.083	30.079 (6.8, 53.4)	0.011
Non-manual workers	0.026	8.985 (-21.5, 39.4)	0.562	-	-	-
Skilled manual workers	0.027	10.769 (-24.4, 45.9)	0.547	-	-	-
Semi-skilled/unskilled workers	0.275	113.8 (78.9, 148.8)	< 0.001	0.253	104.928 (78.3, 131.6)	< 0.001
Urban location	-0.119	-34.170 (-59.1, -9.3)	0.007	-0.065	-18.773 (-36.7, -0.8)	0.041
Supplement users	-0.027	-0.004 (-0.0, 0.0)	0.540	-	-	-
Under reporters	-0.156	-46.241 (-71.8, -20.7)	< 0.001	-0.050	-14.838 (-35.8, 6.1)	0.165
Mean daily energy intake (kcal/day)	0.229	0.083 (0.1, 0.1)	< 0.001	0.101	0.036 (0.0, 0.1)	0.002
Mean daily milk consumption (g/day)	0.642	0.613 (0.5, 0.7)	< 0.001	0.617	0.589 (0.5, 0.7)	< 0.001

Univariate and multiple (multivariable) linear regression analysis examining the associations between sociodemographic characteristics, parental factors, and dietary factors with school-age girl’s iodine intake (µg/d). R²=0.509, adjusted R²= 0.504 (*p* < 0.001). Age reference category = 5–10-year-olds; education reference category = tertiary; social class reference category = professional, managerial & technical workers; location reference category = rural; supplement use reference category = no; underreporting reference category = adequate reporter. β, standardised Beta coefficient; B, unstandardised coefficient; 95% CI, 95% confidence interval (lower, upper); BMI, body mass index; mins, minutes. Lower parental education level refers to secondary, intermediate, and primary education

## Discussion

Overall, the median UIC for the total population suggests mild iodine deficiency in school-age girls living in Ireland, with 40% of this population having intakes of iodine below the EAR. Older girls (11–18 years) had significantly lower iodine intake in comparison to younger girls (5–10 years) (86 µg/d compared to 108 µg/d; *p* < 0.001). Moreover, in this cohort, 25% of 5–10-year-old girls and 32% of 11–18-year-olds did not achieve the LRNI recommendation. Milk consumption and season had a significant association with iodine intake and status, with greater levels of both observed in higher consumers of milk and in those surveyed during the winter months.

The WHO state that a median UIC of 50–99 µg/L suggests mild iodine deficiency and that no more than 20% of the population’s UIC should fall below 50 µg/L [2, 14]. The median UIC for school-aged girls in the current study was 87.8 µg/L, with 18% of girls failing below this threshold. In terms of the Irish population, this is a notable lower UIC compared to studies conducted in Irish adults in 2008-10 [16] and in adolescent females conducted in 2014-15, (107 µg/L and 111 µg/l respectively) [17, 18] and may indicate that the iodine status is declining in the Irish population in more recent years. Other European countries have also reported mild iodine deficiency based on UIC. In Finland, the 2017 national dietary survey revealed that the median UIC for Finnish adults (24–74 years) was 96 µg/L, suggesting mild iodine deficiency [38]. For many years, Finland maintained a successful iodised salt program; however, a decline in the consumption of iodised salt and milk has led to reduced iodine intake [39] and status in certain Finish population groups [40]. In Norway, the iodine status of 403 young Norwegian females (18–30 years) was assessed and the median UIC was 75 µg/L, with 31% of females falling below 50 µg/L [41]. The Norwegian population was classed as iodine sufficient for many years mainly due to high milk intake in the population. However, similar to other countries milk consumption is declining in Norway [41]. A recent cross-sectional national study in Swiss children aged 6–12 years (*n* 362) and pregnant women (*n* 513) found that the median UIC in children remained relatively stable from 1999 to 2022, although it continues to remain at the lower end of the adequate range (127 µg/L). The results also suggest a decline in iodine intake among pregnant women as evidenced by an insufficient median UIC (97 µg/L), particularly in those not consuming iodine-containing supplements (129 vs. 81 µg/L, *p* < 0.001) [42]. At present, iodised salt is only available in the form of household salt in Norway [41]. In Switzerland it is estimated that over 80% of households consume iodised salt [43]. However, just one-third of processed foods containing salt are produced with iodised salt in Switzerland [44]. The recent WHO report on the prevention and control of iodine deficiency in the European region states that foods produced or cooked outside the home, including bread, processed meats, and ready-to-eat meals, now account for 70–80% of total salt intake in a Western diet [14]. While iodised salt is an important dietary source of iodine in the Norwegian and Swiss diet it does not alone meet the dietary requirements and milk and dairy products remain important sources of iodine particularly in children [42, 45].

While the consequences of iodine deficiency for young children are well established, the consequences of mild-moderate iodine deficiency in older children are less known. A double-blinded randomised placebo control study carried out in New Zealand, involving 184 mildly iodine deficient children (median UIC 63 µg/L) from the ages of 10 to 13 years, examined the effect of dietary supplementation with 150 µg per day of iodine as potassium iodate for 28 weeks. The results showed an improvement in iodine deficiency with an optimisation of status and a significantly improved performance in two out of four cognitive tests compared to the placebo group. Furthermore, there was a significant improvement in the total cognitive score by 0.19 standard deviations in comparison to children administered the placebo [46]. Similarly, a double-blinded randomised controlled trial in Albania involving 310 moderately iodine deficient (UIC 42 µg/L) 10–12-year-old children found that compared to the placebo group, supplementation of 400 mg oral iodised oil was positively associated with cognitive processing, fine motor skills, and problem-solving through visual challenges [47]. Both studies indicate that a mild to moderate deficiency may hinder older children from reaching their full intellectual potential. Thus, the identification of mild iodine deficiency in schoolgirls living in Ireland in this current study raises significant public health concern.

Milk provided over 50% of iodine intakes in this population, which is similar to other European surveys in children which showed that milk contributed to 42% of iodine intakes in Germany [48] and dairy products including milk contributed to 44% of iodine intakes in Spain [49] and in the UK, milk and milk products contributed 51% of iodine intakes in children and 40% in adolescents [50]. Milk was positively associated with greater iodine intake and status in this cohort with non-consumers of milk having significantly lower iodine intake and status when compared to high consumers of milk. The same has been observed in other cross-sectional studies, specifically in Ireland and the UK where no iodised salt policy exists [16, 18, 51]. Milk is a good source of iodine as in some regions, it is standard practice for farmers to use iodine-based disinfectants for the animals’ teats and to supplement their livestock with concentrates containing trace minerals or mineral licks. Which is why milk produced in the winter months often contains more iodine than the summer months as cattle are usually housed in the winter and are fed concentrates containing essential trace minerals [52, 53].

In this analysis, dietary and urinary iodine were significantly greater in October to March compared to April to September which is potentially due to milk having higher iodine concentrations in the Autumn-Winter months when animals are housed. Other studies have also observed this seasonal difference [16, 54]. That being said, in some countries the use of iodine-based disinfectants has declined which has resulted in lower iodine concentrations in milk in countries such as Australia and New Zealand [55, 56]. Nonetheless, Australia and New Zealand have implemented mandatory iodine fortification of all bread (excluding organic bread) which has shown to be an effective strategy in increasing iodine intake. Before mandatory fortification in New Zealand, school children aged 5–14 years had a median UIC of 68 µg/L [57] and similarly Australian children aged 8–10 years had a median UIC of 96 µg/L [58], both suggesting mild iodine deficiency. However, since mandatory fortification was introduced in 2009, the median UIC for children (8–10 years) living in New Zealand has increased to 116 µg/L [59] and for children (5–11 years) living in Australia it has increased to 176 µg/L [60], both indicating adequacy. Thus, showing that using bread as a food vehicle to increase iodine intakes in the population seems a promising strategy.

In 1993, the WHO and UNICEF recommended iodisation of salt to combat iodine deficiency and similar to Australia and New Zealand, many countries in Europe have established a fortification programme. Ireland has not introduced such a policy as yet, so the population relies on the current food supply for adequate iodine intakes. Thus, changes in dietary patterns in terms of iodine rich sources should be monitored, as a concern is that with the decline in milk consumption amongst children and adolescents this could have a negative impact [19, 20]. Furthermore, alternative milk drinks options are becoming increasingly more popular but are not always a sufficient substitute for conventional cow’s milk in terms of iodine content [61–64]. As we move towards a more plant-based diet to combat sustainability concerns, iodine intake must be considered as currently the main dietary sources of iodine are animal product based. This is highlighted in a recent narrative review on iodine and plant-based diets, with the researchers indicating that plant-based diets pose the risk of iodine deficiency, particularly in countries such as the UK and Ireland where no iodine fortification program exists [65]. This concern is also raised in the latest WHO report on the prevention and control of iodine deficiency which notes a decrease in cow’s milk and a shift towards plant-based milk alternatives, especially among females [14].

Regarding the factors were associated with iodine intake and status in this cohort, it was evident that age played a significant role. with younger girls having a significantly higher UICs compared to older girls. This is possibly driven by milk consumption which was also significantly lower in the older compared to the younger age group. This relationship between increasing age and declining milk consumption and iodine intakes is in line with other research which found that dietary iodine and status tend to decrease in females from childhood to adulthood, mainly because of changing eating patterns. In the UK, in the NDNS (2016/17-2018/19) 8% of 4–10-year-old girls and a higher percentage of 28% of girls aged 11–18 years failed to achieve the LRNI for their age group [50]. In North-East Italy a study which investigated iodine status from childhood to adulthood showed that milk intake decreased throughout age groups with 76% of young girls reporting consuming one or more cups of milk per day compared to 62% of adolescent girls and 56% of women. Among non-consumers of milk, 56% of children and 81% of women had UICs < 100 µg/L [66]. Given that milk consumption seems to decline as women progress through life and milk is a key source of iodine particularly in countries with no iodised salt programme, these findings are concerning.

Living in a rural area was positively associated with iodine intakes in the current study, which is similar to findings from Norway’s Ungkost 3 national dietary survey (2015–2016) which showed that there was a significant difference in the iodine intakes of children and adolescents from different locations, with children and adolescents living in mid- and western Norway having greater iodine intake in comparison to children and adolescents residing in the capital city and surrounding area [67]. In the current study after further investigation, it was found that those living in a rural location had significantly greater intakes of milk compared to those living in an urban area in this cohort. Thus, milk may be potentially driving this association, however it is unclear why those living rurally have different dietary practices in terms of milk consumption. Therefore, future research is warranted to investigate this observed trend further and possibly review milk consumption patterns. The Ungkost 3 national dietary survey also found that iodine intakes were greater in children and adolescents whose mother had a higher level of education compared to those whose mother had a low level of education [66], which is similar to that found in other studies in school-age children in Spain [68, 69] and the Netherlands [70]. However, this is in contrast to the present study, where a lower level of paternal education was associated with higher iodine intakes in younger girls. In the current analysis, there was no significance difference in milk consumption habits between parental education or social class groups. Foods that were contributing to the higher iodine intakes in girls with parents with a lower parental education and social class included savoury dishes such as pizza, chocolate confectionery and fish and fish dishes including products such as breaded and battered fish. So, although providing increased intake of iodine, the overall foods maybe higher in fat, sugar and salt and therefore would potentially lead to lower diet quality. This unique relationship warrants further investigation to be understood fully.

This study has many strengths, it was nationally representative of the Irish population and provides an insight into iodine intake and status of school-age girls living in Ireland and the factors that were associated with iodine intake and status in this population group. To estimate iodine intake, detailed dietary data collected at brand level, and analytical iodine values were used from commonly consumed products in Ireland for most foods (> 95%) instead of values from standard food composition databases [22]. Furthermore, iodine status was analysed for a subgroup of 496 participants based on UICs. The quantity of iodine in the urine can be evaluated by 24-hour urine collections or by urine spot samples known as urinary iodine concentration (UIC) [71]. It is recommended that UIC from spot samples are used, as obtaining urine spot specimens are a more convenient way of collecting samples as opposed to 24-hour samples which may be non-viable in large population studies [2]. Furthermore, the laboratory used was registered with the Center for Disease Control and Prevention (CDC) standardization program for assessing iodine deficiency recognised as the Ensuring the Quality of Urinary Iodine Procedures (EQUIP) program [72]. To account for the seasonal variance of milk, seasonal values were included based on the season the participant took part in the survey. Moreover, Ireland does not have a salt iodisation programme, and it is uncommon to find iodised salt in Irish supermarkets [73]. Thus, this study provides a reliable estimation of dietary iodine intake. Nonetheless, limitations should be noted, dietary iodine intakes were estimated based on a four-day weighed food diary which introduces the typical issue of reporting bias associated with self-reported data. However, trained researcher interaction was employed to help limit this bias. The urine samples were obtained as first void spot samples which only reflect short term iodine status. It is also important to note the diurnal variation in UICs, with lower levels observed in morning samples [74, 75]. Consequently, the UICs in the present study may be higher due to this diurnal effect. Lastly, only females were considered in this analysis and although it is noted that they are more vulnerable to iodine deficiency, inclusion of males in future research would be important to give an overall picture of iodine status in school-aged children.

Overall, this study indicates that almost half of school-aged girls in Ireland had iodine intakes which fell below the EAR, and the median UIC for the total population indicates mild iodine deficiency. Given the concerns associated with mild iodine deficiencies and their potential implications for public health, these findings highlight the urgency of initiating a large-scale investigation into the iodine status of the Irish population with a particular focus on women of childbearing age or pregnant women as these groups are also classed as vulnerable in terms of iodine deficiency. Dietary patterns and dairy practices may need to be closely monitored especially due to recent declines observed in dairy consumption in this age group. The development of targeted interventions and public health strategies to eradicate iodine deficiency may be warranted such as the consideration of iodised salt fortification or efforts to increase fish and seaweed consumption.

## Electronic supplementary material

Below is the link to the electronic supplementary material.

Supplementary Material 1: Online Supplementary Fig. S1 The relationship between age (years) and mean daily dietary iodine intake (µg/d) and milk consumption (g/d) Milk consumption refers to consumption of whole, semi-skimmed and skimmed milks. Data presented as adjusted means derived from a covariate-adjusted general linear model. Covariates included: household location, education level of parent/guardian, social class, and underreporting.??? Mean values with unlike letters were significantly different across age groups; mean daily dietary iodine intakes (µg/d) (p = 0.022, n^2^p = 0.011), milk consumption (g/d) (p <0.001, n2p = 0.031).
