# Supplementary material for: Iodine intake and status of school-age girls in Ireland
Source: Eur J Nutr. 2025 Jun 9;64(5):214. doi: 10.1007/s00394-025-03731-9 (PMC12148989; doi:10.1007/s00394-025-03731-9)
Supplement: Supplementary file 1 — Supplementary Material 1: Online Supplementary Fig. S1 The relationship between age (years) and mean daily dietary iodine intake (µg/d) and milk consumption (g/d) Milk consumption refers to consumption of whole, semi-skimmed and skimmed milks. Data presented as adjusted means derived from a covariate-adjusted general linear model. Covariates included: household location, education level of parent/guardian, social class, and underreporting.??? Mean values with unlike letters were significantly different across age groups; mean daily dietary iodine intakes (µg/d) (p = 0.022, n2p = 0.011), milk consumption (g/d) (p <0.001, n2p = 0.031). [file 394_2025_3731_MOESM1_ESM.docx]

**Online Supplementary Fig. S1** The relationship between age (years) and mean daily dietary iodine intake (µg/d) and milk consumption (g/d)

Milk consumption refers to consumption of whole, semi-skimmed and skimmed milks. Data presented as adjusted means derived from a covariate-adjusted general linear model. Covariates included: household location, education level of parent/guardian, social class, and underreporting. ᵃ˒ᵇ Mean values with unlike letters were significantly different across age groups; mean daily dietary iodine intakes (µg/d) (*p* = 0.022, n^2^p = 0.011), milk consumption (g/d) (*p* < 0.001, n^2^p = 0.031).
